# High Prevalence of Stunting and Anaemia Is Associated with Multiple Micronutrient Deficiencies in School Children of Small-Scale Farmers from Chamwino and Kilosa Districts, Tanzania

**DOI:** 10.3390/nu13051576

**Published:** 2021-05-08

**Authors:** Victoria Flavian Gowele, Joyce Kinabo, Theresia Jumbe, Constance Rybak, Wolfgang Stuetz

**Affiliations:** 1 Department of Food Technology Nutrition and Consumer Sciences, College of Agriculture, Sokoine University of Agriculture, Morogoro P.O. Box 3006, Tanzania; vgowele@sua.ac.tz (V.F.G.); joyce_kinabo@yahoo.com (J.K.); tjumbe2000@yahoo.com (T.J.); 2 Institute of Nutritional Sciences, University of Hohenheim, 70599 Stuttgart, Germany; 3 Leibniz Centre for Agricultural Landscape Research (ZALF), 15374 Müncheberg, Germany; constance.rybak@zalf.de

**Keywords:** school children, stunting, anaemia, vitamin A, iron, micronutrient deficiency, Tanzania

## Abstract

Inadequate macro- and micronutrient nutrition and its consequences, such as anaemia, iron and vitamin deficiency, and growth retardation, could particularly affect children of small-scale farmers. In the present cross-sectional study, 666 school children aged 5–10 years from villages of Chamwino and Kilosa districts were studied for associations between nutritional and micronutrient status and dietary intake. The overall prevalence of stunting, underweight, and overweight was 28.1, 14.4, and 5%, while that of anaemia and deficiency of iron (ID), vitamin A (VAD), and zinc (ZnD) was 42.9, 29.3, 24.9, and 26.4%, respectively. Dietary recalls (24h) revealed that, except of iron (74%), only small proportions of children reached the recommended daily micronutrient intakes: 4% for zinc, 19% for vitamin A, and 14–46% for B vitamins. Stunting was highly associated with wasting in both districts and with VAD in Chamwino. Anaemia was predicted by ID, VAD, and ZnD in Chamwino and by elevated infection markers, C-reactive protein (CRP) and α-1 glycoprotein (AGP), in Kilosa. Overall, elevated CRP and/or AGP increased the risk while higher serum carotenoids indicating a diet of more fruit and vegetables reduced the risk of VAD. The significantly lower prevalence of anaemia and ID in Chamwino was related to higher iron and vitamin A intake and the consumption of mainly bulrush millet with dark green leafy vegetables compared to maize or rice with legumes in Kilosa. Nutrition and hygiene education integrated with home and school garden programmes could reduce the multiple burdens of anaemia; micronutrient deficiencies and infections; and, in the long term, the prevalence of stunting.

## 1. Introduction

Micronutrient malnutrition, especially of vitamin A, iron, and zinc, are common among children and remains one of the major public health challenges in developing countries [1]. Malnutrition in children can result both from a diet that is poor and deficient in essential macro- and micronutrients, and from the inefficient utilization of available nutrients due to infections and parasitic infestations [2,3].

In rural Tanzania, stunting, anaemia, iron, and vitamin A deficiency are among the most prevalent nutritional problems in school children [4,5,6,7,8]. Studies reported a high prevalence of stunting (21–79%), anaemia (29–80%), and deficiency of iron (33%) and vitamin A (32%), depending on the study area (Northwestern, Central, or Southern Tanzania), the age group of the children, and the year the study was conducted (from 1996 to 2015). However, all these studies as well the Tanzanian national demographic, health, and nutrition surveys [9,10], missed a detailed nutritional assessment regarding micronutrient intake and status; further, the national surveys only focused on young children below 5 years of age, but malnutrition persists into the preadolescent period affecting school performance and reproductive health during puberty, particularly when child-bearing occurs early in life [11].

Stunting is a form of chronic malnutrition that reflects failure to receive adequate nutrition over a long period due to poor diets, recurrent infections, and chronic illness [12]. The long-term effects of stunting in individuals includes diminished cognitive and physical development; reduced productive capacity; and increased risk of degenerative diseases, such as diabetes. In addition, stunted children experience rapid weight gain and thus have an increased risk of becoming overweight or obese later in life [13].

Anaemia remains a major public health challenge among school children in Tanzania where infectious diseases, such as malaria and soil-transmitted helminths (STH), are highly prevalent [4,6]. Micronutrient deficiencies, especially of iron, vitamin A, folate, and vitamin B12, usually occur simultaneously and in conjunction with micronutrient-poor diets, which then leads to anaemia and other deficiency symptoms via synergistic effects [14,15].

The present study is part of the Scale-N project [16] aiming to achieve food and nutrition security of small-scale farmers in Dodoma and Morogoro regions by the support and development of nutrition-sensitive agricultural production, the establishment of pocket gardens for the production and consumption of leafy vegetables, and the improvement of nutritional behaviour [17]. At the baseline study in 2016, school children (in pre- and primary school aged 5–10 years) of four different rural villages in the Chamwino and Kilosa districts were enrolled along with their mothers to study (1) their nutritional and micronutrient status; (2) dietary intake in regard to the adequacy of micronutrients; and (3) determinants of stunting, wasting, anaemia, and iron- and vitamin A deficiency.

## 2. Materials and Methods

### 2.1. Study Area and Population

This cross-sectional survey was part of the baseline study under the Scale-N project conducted during the period July–August 2016 in Dodoma and Morogoro regions, Tanzania. Two districts in the regions of Dodoma and Morogoro were purposively selected due to their high prevalence of anaemia in children aged 6–59 months, as shown in the national ‘Tanzania Demographic and Health Survey’ [10]. The semi-arid Chamwino district (350–500 mm annual rainfall) is one of seven districts of the Dodoma region and consists of primarily flat plains; the predominantly sub-humid Kilosa district (600–800 mm annual rainfall) is one of seven districts of the Morogoro region and is characterized by flat plains, highlands, and dry alluvial valleys. Briefly, the food system in Chamwino is primarily based on millet and sorghum with a deep attachment to livestock [18,19]. Other crops commonly cultivated include sunflower, groundnuts, and bambara nuts. In the Kilosa district, legumes, rice, sorghum, maize, and horticultural crops are common, with some livestock integrated into the livelihood system [18].

The households were sampled from four villages participating in the Scale-N project, Mzula and Chinoje in the Chamwino district, and Tindiga and Mhenda-Kitunduweta in the Kilosa district. The villagers and study population were practically all self-sufficient small-scale farmers. The original plan was to include at least 150 households with associated school-age children (6–9 years) in each of the 4 villages. More than 165 households per village with the inclusion criterion of having a mother and a corresponding school-age child were randomly assigned from the village register (using ENA for SMART software version 2011). A total of eligible 669 households with mothers or caregivers and apparently healthy school children aged 5 to 10 years were successfully enrolled in the study. The survey was carried out according to the guidelines laid out in the Declaration of Helsinki and approved by the National Institute for Medical Research and the Ministry of Health, Community Development, Gender, Elderly and Children in Dar es Salaam, Tanzania (NIMR/HQ/R.8a/Vol. IX/2226) and the Ethics Committee Landesärztekammer Baden-Württemberg, Stuttgart, Germany (F-2016-049). Written informed consent was obtained from 669 mothers and/or caregivers for the analysis of nutritional status (anthropometry and dietary intake) and the collection of blood for the determination of serum micronutrients; three households with mother–child pairs were excluded from the analysis due to missing blood draw or uncompleted questionnaires, giving a total sample size of 666 school children within the baseline survey.

### 2.2. Socio-Demographic Information

Socio-demographic information (personal data) and dietary intake patterns of the study population were collected using the pre-tested structured Scale-N project survey tool. The questionnaire was interview based and prepared in both English and Swahili version, while the Swahili version was the one posed during the interview. All enumerators including those who took the anthropometric measurements received intensive training prior the survey following the guidelines endorsed by FANTA III, Training Package for Facility-Based Service Providers. The questionnaire also included questions about mother/caregiver literacy; household size; and information on the child’s age, sex, and reported malaria (in the last 3 months) and diarrhoea (in the last 4 weeks).

### 2.3. Anthropometric Assessment

Body weight was measured in light clothing to the nearest 0.1 kg using a SECA electronic scale with a tare facility (SECA GmbH & co.kg, Hamburg, Germany). Height was measured to the nearest 0.1 cm using a stadiometer (Model S0114540, UNICEF, New York, NY, USA). The height measurement was taken while the child was standing without shoes on a horizontal flat plate attached to the base of the stadiometer with heels together, and stretched upwards to the full extent with the head looking straight ahead. The mid upper arm circumference (MUAC) between the acromion and the olecranon process (shoulder and elbow) was measured to the nearest 0.1 cm using standard MUAC measuring tapes for reference age (UNICEF). Anthropometric indices, height-for-age (HAZ), weight-for-age (WAZ), and body mass index-for-age (BMIA) Z-scores were computed using WHO AnthroPlus (v1.0.4) software; nutritional status indices for overweight (>+1SD BMI-for-age Z score), obesity (>+2SD BMI-for-age Z score), thinness/wasting (<−2SD of BMI-for-age Z score), underweight (<−2SD of weight-for-age Z score), and stunting (<−2SD of height-for-age (HAZ) Z score) were defined according to the WHO reference growth charts for children aged 5 to 19 years [20].

### 2.4. Dietary Assessment

A single 24 h dietary recall method was applied to assess the dietary intake of the target child. We calculated the 24 h macro- and micronutrient intake (vitamins and minerals) of all reported foods per amount (grams and litres) for each child using the ‘NutriSurvey’ software package [21]. This software contains all reported foods and recipes listed in the Tanzanian food composition tables [22], and, additionally, the micronutrient contents of indigenous leafy vegetables collected in the study area [23] were entered and processed. We analysed the adequacy of micronutrient intake using the recommended nutrient intakes (RNIs) by WHO [24]. For macronutrients, the percentage of total energy intake from proteins, carbohydrates, and total fat was evaluated using the following acceptable macronutrient distribution ranges (AMDR) for children aged 4 to 18 years: 10–30% for protein, 25–35% for fat, and 45–65% for carbohydrate [25].

### 2.5. Blood Sampling and Analysis

Venous blood (3–5 mL) was drawn from each target child using sterile safety multifly needles (butterfly) and serum monovettes. Haemoglobin (Hb) concentrations were measured immediately on site by transferring a drop of venous blood taken from the sterile safety multifly into microcuvettes and measuring it with a portable battery-operated haemoglobinometer (HemoCueHb 201+, Angelhom, Sweden). Anaemia was defined as Hb < 115 g/L for children aged 5–11 years [26].

Venous blood samples were centrifuged at 1850× *g* for 15 min at room temperature, and serum aliquots were distributed and transferred to 2.0 mL Eppendorf tubes, frozen at −20 °C at study sites, and further transported to the laboratory at Sokoine University of Agriculture (SUA) in Morogoro for storage at −80 °C, before finally being transported on dry ice to the University of Hohenheim, Stuttgart, for the analysis on serum micronutrients. Retinol (vitamin A), carotenoids, and tocopherols (vitamin E) were determined using high-performance liquid chromatography (HPLC), while serum aliquots were analysed (at the VitMin Lab, Dr. JG Erhardt, Willstaett, Germany) on iron status markers (ferritin; sTfR, soluble transferrin receptor), infection/inflammation markers (CRP, C-reactive protein; AGP, α-1 glycoprotein) by a sandwich enzyme-linked immune-sorbent assay technique, and serum zinc by a spectrophotometric method as previously described in detail [17,27]. Iron deficiency (ID) was defined as ferritin < 15 µg/L or sTfR > 8.5 mg/L for children >5 years [28], while total body iron stores (IST) were calculated by an equation using ferritin and sTfR [29]. Elevated acute phase proteins CRP > 5 mg/L and AGP > 1 g/L were used as indicators for an acute phase response by infection or inflammation [30], and serum ferritin was adjusted for respective correction factors for children and the 3 different inflammation stages: factor 0.64 for incubation (CRP > 5 mg/L and AGP ≤ 1 g/L), 0.39 for early convalescence (CRP > 5 mg/L and AGP > 1 g/L), and 0.65 for late convalescence (CRP ≤ 5 mg/L and AGP > 1 g/L). Retinol < 0.7 µmol/L was considered to be indicative of vitamin A deficiency (VAD) [31], while serum zinc < 0.65 mg/L was used to indicate low/deficient zinc status for children <10 years [32]; serum zinc was not adjusted according to the BRINDA analysis study.

### 2.6. Statistical Analysis

Socio-demographic characteristics, anthropometrics (weight), blood biomarkers (haemoglobin, acute phase proteins, and serum micronutrients), and data on the dietary intake of micronutrients of the study participants are described using medians with interquartile range (IQR), mean (SD), and frequencies (number), as appropriate. For comparisons between the study villages and districts, the Kruskal–Wallis, Mann–Whitney U-test, and ANOVA (post hoc Scheffe test) for continuous variables and Chi-squared tests for prevalence and categorical data were used. Multiple logistic regression analysis with a forward (stepwise) approach was applied to identify independent predictors of stunting (HAZ < −2SD), underweight (WAZ < −2SD), overweight (BMI > 1SD), anaemia (haemoglobin < 115 g/L), iron deficiency (ferritin < 15 µg/L or sTfR > 8.5 mg/L), and vitamin A deficiency (serum retinol < 0.7 µmol/L) in the two districts. The following co-variables were assessed in the initial and separate models: age, sex, reported malaria (in the last 3 months) or diarrhoea (in the last 4 weeks), family size (number of persons in the HH), mother’s literacy status (can read and write vs. not), accompanying malnutrition (HAZ < −2SD, WAZ < −2SD, BMI > 1SD), anaemia (yes = 1 vs. no = 0), MN deficiencies (of iron, vitamin A, or zinc; yes = 1 vs. no = 0), serum micronutrients (e.g., β-carotene), elevated acute phase proteins CRP or AGP, and sufficient dietary intakes of assessed MNs (e.g., iron or vitamins ≥ RNI); probability for the stepwise entry and removal were set at *p* < 0.05 and *p* < 0.10, respectively; appropriate fit of the logistic regression models was confirmed using the Hosmer–Lemeshow goodness-of-fit test. All statistical analyses were carried out using SPSS software (SPSS Inc., Chicago, IL, USA; Version 20.0.0), and *p* values < 0.05 were considered as statistically significant.

## 3. Results

Socio-demographic characteristics, anthropometry, and morbidity data of children were compared by village and are summarized in Table 1. The study included a total of 666 children (54.7% females) with a median (IQR) age of 7.3 (6.4, 8.1) years; almost every second mother and/or caregiver (43%) could not read and write. The overall stunting prevalence was 28%, ranging from 25% in Chinoje to 35% in Mhenda-Kitunduweta village; the prevalence of underweight was 14%, ranging from 11% in Tindiga to 17% in Mzula village; overweight was more frequent in the Kilosa villages (7–8%) than in the Chamwino villages (2.4%). In agreement, the median MUAC and BMI-for-age Z-scores were significantly higher in Kilosa district than in Chamwino district. The highest proportion of overweight (7.9%) and stunting (34.8%) was in the Mhenda-Kitunduweta village, where the children had simultaneously the highest prevalence of reported malaria and diarrhoea in the weeks and months prior to the survey.

The results of haemoglobin (Hb) and iron status (ferritin, sTfR), serum micronutrients (retinol, zinc, carotenoids, and tocopherols), and infection markers (CRP, AGP) by village are presented in Table 2. The median (IQR) haemoglobin was 116 (109, 125) g/L, and 42.9% of all children were anaemic (Hb < 115g/L). Children from villages in Kilosa district had significantly lower haemoglobin than those in Chamwino, and anaemia prevalence was more than twice as high (59.2 and 68.3%) than in children from villages in the Chamwino district (18.6 and 25.9%). The overall prevalence of elevated acute phase proteins was 11.7% for CRP and 22.3% for AGP. Children from Mhenda-Kitunduweta (Kilosa) had the highest and also a significantly higher prevalence of increased CRP and AGP compared to the other villages; simultaneously, children from this village had the highest median serum ferritin concentration, even after adjustment for stages of inflammation, but the highest median soluble transferrin receptor (sTfR). indicating tissue iron deficiency; Mhenda-Kitunduweta showed, excluding Tindiga (another village in Kilosa), the highest prevalence of iron deficiency (ID) among the four study villages. The overall prevalence of ID was 29.3%, and in the villages of Kilosa was significantly higher than in those from Chamwino (39.1 and 40.2% vs. 20.4 and 17.6%). The median serum zinc concentration was 0.723 mg/L, with 26.4% of all children and 39% of children from Chinoje showing deficiency (ZnD) or low serum values of zinc (<0.65 mg/L).

Median serum retinol of all children was 0.853 µmol/L, and each fourth child had vitamin A deficiency (24.9% < 0.7 µmol/L); as with zinc, Chinoje was the village with the highest burden, with one in three children suffering from VAD (34.3%). The serum concentration of γ-tocopherol was higher, while α-tocopherol was lower in Chamwino than in Kilosa. The differences in carotenoids between the districts and individual villages were as follows: regarding pro-vitamin A carotenoids, α-carotene was higher in Kilosa than in Chamwino, β-carotene was similar in the villages except for higher concentrations in Chinoje, while β-cryptoxanthin was significantly higher in Mzula and Mhenda-Kitunduweta than in the other villages. Lutein-zeaxanthin was overall about twice as high, while lycopene was lower in Chamwino than in Kilosa villages.

The assessed macro- and micronutrient intakes by 24 h recalls are presented in Table 3. The minimum recommended energy intake for the respective age and sex categories was overall only reached by 10% of the surveyed children; the protein consumption was at the lower limit, fat was below the lower limit, while carbohydrate intake exceeded the upper limit of the acceptable macronutrient distribution ranges of energy intake.

Children from Chamwino had a significantly lower intake of energy, protein, fat, and carbohydrates but at the same time a significantly higher intake of vitamin A, calcium, and iron than those from Kilosa. However, only 19, 26, and 74% of the children reached the recommended nutrient intake (RNI) for vitamin A, calcium, and iron, respectively. The intake of vitamin E was in particular low, with only 5% reaching the RNI. In Kilosa, the intake of almost all B-vitamins tended to be higher and of ascorbic acid (AA) was significantly higher than in the Chamwino villages; Mhenda-Kitunduweta was the village with the highest intake of B-vitamins and AA; however, on average, only each fourth child reached the RNI for B1, B2, or AA, while only 14, 46, and 34% reached the RNI for B12, B6, and folate, respectively.

Calculated mineral intake regarding zinc was similarly low and of magnesium similarly high between the districts and villages; but only 5% reached the RNI for zinc, while three of four children were able to achieve the RNI of magnesium. In summary, energy and fat intake was too low, and vitamin E, zinc, and vitamin B12 were the most limiting micronutrients in the diets of studied children.

The micronutrient status and intake and frequency and quantities of consumed food items in the two districts are summarized in Table 4. Children from Chamwino had a clearly and significantly lower prevalence of anaemia, ID, and of elevated infection markers CRP or AGP, but a higher prevalence of VAD and ZnD than those from the Kilosa villages; further, serum β-carotene, lutein-zeaxanthin, and γ-tocopherol were higher, while α-carotene, lycopene, and α-tocopherol were lower in Chamwino than in Kilosa children. The results of biological markers were largely consistent with the calculated MN intakes: the intake of iron and RE was twice as high, while that of zinc and vitamin E was lower in Chamwino than in Kilosa; however, the supply of zinc and vitamin E was in both districts clearly insufficient (<5% reached the RNI). In Chamwino, children consumed most frequently dishes that comprised bulrush millet and dark green leafy vegetables (DGLV). In Kilosa, the more varied diet consisted of maize or rice in combination with higher quantities of either legumes, roots, or other vegetables compared to Chamwino. Fruits, meat, and fish were more frequently consumed in Kilosa, but overall, in both districts, it was rare (2–25%) or even the exception.

Multiple logistic regression models to identify factors that are significantly associated with (1) stunting, underweight, and overweight, and with (2) anaemia, ID, and VAD are summarized in Table 5 and Table 6. In both districts, stunting was highly associated with underweight (odds ratio of 24 and 29) and in Chamwino with VAD and younger age. In both districts, older and stunted children were at higher risk, while increasing MUAC was associated with a lower risk of underweight; in agreement with this, MUAC was positively associated while age was inversely associated with overweight. Notable, the positive association of overweight with ZnD in Chamwino, and the higher risk of overweight with ID, serum lutein-zeaxanthin, and higher vitamin B6 intake in Kilosa (Table 5).

ID, VAD, ZnD, and sufficient vitamin B6 intake (intake ≥ RNI) were associated with anaemia in Chamwino; elevated acute phase proteins (CRP or AGP) were positively while serum lycopene was inversely associated with anaemia in Kilosa (Table 6). VAD and reported malaria in Chamwino and CRP/AGP in Kilosa predicted ID. In both districts, elevated CRP/AGP increased the risk whereas serum α-tocopherol reduced the risk of VAD; moreover, serum β-carotene and β-cryptoxanthin in Chamwino and higher serum lutein-zeaxanthin in Kilosa reduced the risk of VAD.

## 4. Discussion

The present study clearly showed that school children in the districts of Chamwino and Kilosa, Tanzania, are simultaneously affected by undernutrition; anaemia; infections, such as malaria; micronutrient deficiencies; and inadequate diets. Our results also indicate significant variations in micronutrient status and dietary habits between districts.

The overall prevalence rates of stunting (28%) and VAD (25%) were of moderate while of anaemia (>40: 22% in Chamwino and 64% in Kilosa) was of severe public health significance [12]. Stunting was in both districts highly associated with underweight and in Chamwino with younger age and VAD. Almost one in two of the stunted children in Chamwino (41/85) and Kilosa (40/102) had simultaneously underweight. This concurrent relationship shows that stunted children could simultaneously experience underweight and/or wasting (low BMI for age), and some children might experience all three forms of anthropometric failures [34], as occurred for one child in this study.

In Chamwino, the decrease in stunting prevalence with age, which is consistent with the overall decremental trend of a younger cohort of children (1–15 years) in Ethiopia [35], suggests both a higher prevalence in early childhood (<five years) and future improvement and catch-up growth. The association between VAD and stunting, in agreement with a study among preschool children in Uganda, may reflect the impact of low vitamin A status over a prolonged period due to both deficient diet and also infections (e.g., diarrhoeal diseases) on growth retardation [36]. Among the four study villages, Mhenda-Kitunduweta (Kilosa) was the village with the highest prevalence of stunting (34.8%), elevated acute phase proteins (40% with high CRP or AGP), and reported recent infection diseases (26% with diarrhoea, and 51% with malaria). The high prevalence of elevated CRP/AGP and reported malaria and diarrhoea suggest persistent and/or recurrent infections, which together with other parasitic worm diseases (i.e., helminths) lead to anaemia and micronutrient deficiencies (ID, VAD, and ZnD) and growth retardation [37]. In Tanzania and Ethiopia, stunting (and wasting) and anaemia are highly prevalent in environments that are characterized by a high prevalence of infectious diseases [5,6,7,38].

Apart from infections, inadequate nutrition itself with the resulting micronutrient deficiencies and anaemia can be assumed to be the decisive cause of stunting of the children in the Scale-N project. In Chamwino, multiple MN deficiencies (ID, VAD, and ZnD) and reported malaria were associated with anaemia, ID, and VAD; elevated CRP/AGP (inflammation) predicted VAD in Chamwino and anaemia, ID, and VAD in Kilosa. Further, ZnD was associated with VAD in Kilosa, while serum carotenoids and α-tocopherol reduced the risk of VAD in both districts. Overall, in Chamwino MN deficiencies whereas in Kilosa ‘infections’ seemed to be mainly responsible for anaemia, ID, and VAD. Further, the calculated intake of iron and vitamin A was much higher and the prevalence of anaemia, ID, and elevated CRP/AGP was much lower in Chamwino than in Kilosa. Higher iron and vitamin A intake in Chamwino was due to the fact that the main local diet consisted of wholemeal millet together with cooked indigenous dark green leafy vegetables (DGLV), as opposed to porridge of polished maize flour or rice combined with either legumes, roots, or other vegetables in Kilosa, as we recently described for the mothers in the Scale-N project [17]. The high intake of DGLV, such as the vegetable dish called ‘Ilende’ [23] made from a locally collected wild leafy vegetable (*Ceratotheca sesamoides*), sundried, and cooked with water and peanuts, is reflected by the significantly higher serum β-carotene and lutein-zeaxanthin than in Kilosa children. However, DGLV and other vegetables were also consumed in Kilosa, reflected in serum carotenoid concentrations [39], e.g., lutein-zeaxanthin (DGLV) or lycopene (tomato and products), and were associated with a reduced risk of anaemia and VAD. Serum lutein-zeaxanthin is apparently derived from DGLV, such as the vegetable dish ‘Mlenda mgunda’ (*Corchorus trilocularis*) [23], while serum lycopene indicates tomatoes as a main ingredient of various vegetable dishes, fresh vegetable salads (‘kachumbari’), and cooked appetizing relishes (‘chachandu’).

It is surprising that the higher consumption of DGLV in Chamwino with correspondingly much higher calculated intake of RE and also slightly higher serum carotenoids did not lead to higher serum retinol (vitamin A status). This may reflect the overall limited consumption of preformed vitamin A food sources, such as meat, fish or eggs [40], as well as insufficient dietary fat intake. The food matrix, processing, and a minimum of dietary fat (3–5 g per meal) are responsible predictors for the absorption of carotenoids from vegetables [41]; the increased consumption of DGLV with oil, which improves bioavailability, was correlated with high plasma retinol among pregnant Tanzanian women [42], and bean and tomato stews with green leafy vegetable powder (GLVP) from eggplants and amaranthus leaves consumed with groundnut soup were more effective than without GLVP in reducing vitamin A deficiency and anaemia prevalence among school children (aged 4–9 years) in a Ghanaian school feeding program [43]. However, as our calculations showed, much higher amounts of MN-rich foods, including DGLV, vegetables, fruits, and meat or fish, are required to reach the RNI for minerals and vitamins according to the WHO [24]. In addition to inadequate vitamin A intake (19% overall achieved the RNI), zinc was, in particular, lacking in the diets of both districts, with only 31 of all children (4.6%) reaching the RNI of zinc. The high prevalence of ZnD (in both the diet and serum) and its association with anaemia and VAD indicate a limited diet of animal origin; poorly available MNs from plant food; and interactions among zinc, VA, and other MNs. In malnourished populations, ZnD often coexists with VAD; zinc is required in mobilizing VA within cells and from the liver through the synthesis of retinol-binding protein (RBP), while VAD may reduce the absorption and transport of zinc by altering zinc by zinc-dependent binding protein [44]. Only β-carotene supplementation along with zinc was able to improve the vitamin A status of both mothers and infants 6 months postpartum, indicating the importance of zinc in relation to VA status in a ‘zinc-deficient’ population [45].

Further, VAD-impaired iron metabolism in cell cultures, and supplementation with vitamin A alone, already showed a reduced risk of anaemia via higher haemoglobin and ferritin in humans with VAD [46,47]. Therefore, a population with ID, VA, or ZnD that relies on a plant-based diet is recommended to increase the consumption of MN-dense food (β-carotene-rich fruits and vegetables) and the bioavailability of the vital nutrients, iron, β-carotene, and zinc through ideal combinations; mild cooking (with fat); and household practices, such as sprouting, fermentation, or the addition of food acidulants and spices [48,49,50]. A vitamin C-rich diet could increase the absorption of iron and thus reduce iron deficiency and the prevalence of anaemia. A recent controlled intervention investigating the impact of baobab fruit pulp (*Adansonia digitata* L.) on the haemoglobin and iron status of school children (aged 6–12 years) in Kenya yielded promising results [51]: the provision of a daily drink containing vitamin C-rich baobab fruit pulp (BFP) for 83 days along with standardized school meals improved haemoglobin concentrations compared to an isoenergetic drink without BFP. The authors concluded that the consumption of foods such as BFP could help improve non-heme iron absorption in populations at risk of iron deficiency, particularly in food-insecure areas where baobab is native, available, and affordable.

Furthermore, malaria and diarrhoeal infections [52,53], as reported in Chamwino and even more so in Kilosa, as well as parasitic worm infection, especially hookworms, as previously described in relation to anaemia in school children in Zanzibar and Lindi, Tanzania [8,54,55], were very likely the constant ‘companions’ and contributing factors to anaemia, ID, and VAD [56,57,58,59]. In addition, the lack of clean drinking water and poor sanitation and hygiene (WASH) are also very likely responsible for the high prevalence of diarrhoeal diseases [52]. Therefore, programs to reduce the prevalence of diarrhoea and parasitic infection as well as those to improve nutritional behaviour (e.g., increased consumption of fruits and vegetables) and food processing are urgently needed to reduce the high prevalence of anaemia, MN deficiencies, and finally stunting in the present study population.

Limitations of the study include the cross-section design with the use of one-time 24 h recalls, blood sampling, and anthropometric measurements, which may reflect the long-term situation to a limited extent; moreover, we did not confirm blood samples for malaria parasites and stool samples for STH infections known to be highly associated with anaemia and ID. In contrast, the strengths of this study include the large sample size with children from two different agro-ecological zones and extensive assessments that were able to indicate and confirm links among inadequate nutrient intake, MN deficiencies, and infections (through biochemical markers) and malnutrition (e.g., stunting).

## 5. Conclusions

In summary, our findings emphasize the high prevalence of stunting and anaemia and their associations with MN deficiencies, infections, and inadequate intakes of essential MNs (e.g., iron, vitamin A, and zinc) among Tanzanian school children of farming communities. Therefore, in a first step, the introduction of home and school gardens integrated with the provision of nutrition and health education are promising strategies to reduce the high burden of anaemia, MN deficiencies, and finally undernutrition. In parallel, programmes to reduce ‘preventable’ parasitic infectious diseases and to improve hygiene (sanitation) should be urgently launched.

## Figures and Tables

**Table 1:** Socio-demographic characteristics and anthropometrics of the children by village.

Village	All	Mzula	Chinoje	Tindiga	Mhenda-Kitunduweta	*p*
District		Chamwino		Kilosa		
Children, N	666	167	166	169	164	
Age, years ^1^	7.3 (6.4, 8.1)	7.4 (6.5, 8.1)	7.1 (6.4, 8)	7.2 (6.4, 8)	7.3 (6.4, 8.3)	0.488
5y (4.6–5.9y) ^2^	12.0 (80)	9.0 (15)	12.7 (21)	14.2 (24)	12.2 (20)	
6y (6.0–6.9y)	30.0 (200)	27.5 (46)	31.3 (52)	30.2 (51)	31.1 (51)	
7y (7.0–7.9y)	27.8 (185)	30.5 (51)	28.9 (48)	29.0 (49)	22.6 (37)	0.196
8y (8.0–8.9y)	19.7 (131)	20.4 (34)	20.5 (34)	13.0 (22)	25.0 (41)	
9y (9.0–10.1y)	10.5 (70)	12.6 (21)	6.6 (11)	13.6 (23)	9.1 (15)	
Sex, female = 1	54.7 (364)	53.9 (90)	53.0 (88)	58.0 (98)	53.7 (88)	0.791
Family size	5 (4, 7)	5 (4, 7) ^a^	6 (5, 7) ^b^	5 (4, 6.5) ^a^	5 (4, 7) ^a^	0.037
Mother literate, =1	56.9 (379)	55.1 (92)	56.0 (93)	52.1 (88)	64.6 (106)	0.117
HAZ ^3^	−1.42 ± 1.05	−1.39 ± 1.0	−1.32 ± 1.10	−1.37 ± 1.06	−1.60 ± 1.03	0.089
Stunting	28.1 (187)	25.7 (43)	25.3 (42)	26.6 (45)	34.8 (57)	0.181
WAZ	−1.11 ± 0.95	−1.25 ± 0.93	−1.09 ± 1.03	−1.0 ± 0.94	−1.09 ± 0.91	0.099
Underweight	14.4 (96)	16.8 (28)	13.9 (23)	11.2 (19)	15.9 (26)	0.485
BAZ	−0.28 ± 0.78	−0.52 ± 0.73 ^c^	−0.31 ± 0.88 ^b,c^	−0.18 ± 0.73 ^a,b^	−0.08 ± 0.71 ^a^	<0.001
Wasting/Thinness	0.8 (5)	0.0 (0) ^a^	3.0 (5) ^b^	0.0 (0) ^a^	0.0 (0) ^a^	0.002
Overweight	5.0 (33)	2.4 (4) ^a^	2.4 (4) ^a^	7.1 (12) ^b^	7.9 (13) ^b^	0.007
Obese	0.2 (1)	0 (0)	0.6 (1)	0 (0)	0 (0)	0.389
Malaria, =1	30.7 (205)	16.7 (28) ^a^	21.1 (35) ^a^	34.9 (59) ^b^	50.6 (83) ^c^	<0.001
Diarrhoea, =1	20.7 (138)	18.5 (31)	22.9 (38)	16.0 (27)	25.6 (42)	0.127

Data are median (25th and 75th percentile) ^1^, percentage (number) ^2^, mean (SD) ^3^, and all such values. *p* values: Kruskal–Wallis, Chi-square test (prevalence) or One-Way ANOVA as appropriate. Villages not sharing a superscript letter (^a,b,c^) are significantly different (*p* < 0.05) to each other (Mann–Whitney U and Tukey HSD tests for continuous variables and Chi-square test for prevalence). MUAC, mid-upper arm circumference; HAZ, height-for-age Z-score; stunting, HAZ< −2SD; WAZ, weight-for-age Z-score; underweight, WAZ < −2SD; BAZ, BMI-for-age Z-score; wasting/thinness, BAZ< −2SD; overweight, 1SD < BAZ ≤ 2SD; obese, BAZ > 2SD [20]; malaria, reported in the last 3 months and diarrhoea, reported in the last 4 weeks.

**Table 2:** Haemoglobin, infection (CRP and AGP) and iron status markers (ferritin and sTfR), and serum micronutrients in children by village.

Village	All	Mzula	Chinoje	Tindiga	Mhenda-Kitunduweta	*p*
District		Chamwino		Kilosa		
Children, N	666	167	166	169	164	
Haemoglobin (g/L) ^1^	116 (109, 125)	126 (117, 132) ^a^	120 (114, 126) ^b^	112 (107, 119) ^c^	110.5 (102, 117) ^d^	<0.001
Hb < 115 g/L, % (n) ^2^	42.9 (286)	18.6 (31) ^a^	25.9 (43) ^a^	59.2 (100) ^b^	68.3 (112) ^b^	<0.001
CRP >5 mg/L	11.7 (78)	6.0 (10) ^a^	9.1 (15) ^a^	10.1 (17) ^a^	22.0 (36) ^b^	<0.001
AGP > 1 g/L	22.3 (148)	10.8 (18) ^a^	12.7 (21) ^a^	28.4 (48) ^b^	37.2 (61) ^b^	<0.001
Ferritin (µg/L)	39.9 (28.5, 59.5)	37.7 (28.8, 56.4) ^a^	36.3 (26.9, 55.2) ^a^	33.7 (24.3, 47.1) ^b^	53.45 (35.1, 82.2) ^c^	<0.001
Ferritin, adj. (µg/L)	34.2 (24.7, 52.6)	36.3 (26.4, 54.4) ^a,c^	34.4 (24.9, 53.8) ^a^	28.0 (20.8, 41.9) ^b^	42.6 (29.5, 57.6) ^c^	<0.001
sTfR (mg/L)	7.17 (6.14, 8.65)	6.76 (5.72, 7.95) ^a^	6.64 (5.79, 7.75) ^a^	7.75 (6.56, 9.50) ^b^	7.93 (6.63, 10.29) ^b^	<0.001
ID, adjusted	29.3 (195)	20.4 (34) ^a^	17.6 (29) ^a^	39.1 (66) ^b^	40.2 (66) ^b^	<0.001
IST, adj. (mg/kg BW)	4.09 (2.55, 5.69)	4.79 (3.01, 6.10) ^a^	4.51 (3.14, 6.10) ^a^	3.16 (1.60, 4.68) ^b^	4.15 (2.81, 5.64) ^a^	<0.001
Zinc (mg/L)	0.723 (0.64, 0.80)	0.752 (0.68,0.84) ^a^	0.666 (0.60, 0.75) ^b^	0.723 (0.65, 0.80) ^a,c^	0.723 (0.64, 0.80) ^c^	<0.001
Zinc < 0.65 mg/L	26.4 (176)	16.8 (28) ^a^	39.2 (65) ^b^	23.1 (39) ^a,c^	26.8 (44) ^c^	<0.001
Retinol, µmol/L	0.853 (0.70, 0.99)	0.848 (0.72, 0.99) ^a^	0.814 (0.63, 0.97) ^b^	0.870 (0.75, 1.00) ^a^	0.878 (0.69, 0.99) ^a,b^	0.011
Retinol, < 0.7 µmol/L	24.9 (166)	22.8 (38) ^b^	34.3 (57) ^c^	16.0 (27) ^b^	26.8 (44) ^a,c^	0.001
γ-Tocopherol, µmol/L	0.706 (0.42, 1.18)	1.108 (0.83, 1.51) ^a^	1.214 (0.81, 1.84) ^a^	0.429 (0.28, 0.63) ^b^	0.456 (0.35, 0.64) ^c^	<0.001
α-Tocopherol, µmol/L	15.36 (13.2, 8.0)	14.45 (12.6, 16.3) ^a^	13.88 (12.0, 15.8) ^b^	17.57 (15.2, 20.3) ^c^	16.26 (14.2, 18.7) ^d^	<0.001
α-Carotene, µmol/L	0.110 (0.04, 0.29)	0.043 (0.03, 0.07) ^a^	0.034 (0.02, 0.05) ^b^	0.286 (0.19, 0.47) ^c^	0.285 (0.18, 0.44) ^c^	<0.001
β-Carotene, µmol/L	0.483 (0.33, 0.68)	0.484 (0.34, 0.67) ^a^	0.554 (0.41, 0.76) ^b^	0.434 (0.30, 0.63) ^a^	0.451 (0.32, 0.64) ^a^	<0.001
β-Cryptoxanthin, µmol/L	0.162 (0.07, 0.34)	0.334 (0.17, 0.51) ^a^	0.077 (0.05, 0.13) ^b^	0.095 (0.05, 0.23) ^b^	0.240 (0.13, 0.50) ^a^	<0.001
Lutein/zeaxanth., µmol/L	0.917 (0.62, 1.24)	1.176 (0.94, 1.50) ^a^	1.174 (0.94, 1.52) ^a^	0.689 (0.50, 0.96) ^b^	0.648 (0.51, 0.83) ^b^	<0.001
Lycopene, µmol/L	0.334 (0.19, 0.57)	0.228 (0. 14, 0.35) ^a^	0.227 (0.15, 0.35) ^a^	0.650 (0.49, 0.87) ^b^	0.378 (0.21, 0.58) ^c^	<0.001

All values are median (25th and 75th percentile) ^1^ or percentage (number) ^2^, all such values; *p*-values: Kruskal–Wallis test for continuous variables and Chi-square test for prevalence. Villages not sharing a superscript letter (^a,b,c,d^) are significantly different (*p* < 0.05) to each other (Mann–Whitney U test, Chi-square test, as appropriate). CRP, C-reactive protein; AGP, α-1 glycoprotein; sTfR, soluble transferrin receptor; ID, iron deficiency, if serum ferritin (adjusted) < 15 µg/L or sTfR > 8.5 mg/L; IST, total body iron stores.

**Table 3:** Calculated macro- and micronutrient intake from 24 h recalls of children by village.

Village	All	Mzula	Chinoje	Tindiga	Mhenda-Kitunduweta	RNI/AMDR
District		Chamwino		Kilosa		
Children, N	666	167	166	169	164	
Energy, EN (Kcal) ^1^	898 (579, 1296)	715 (469, 1003) ^a^	550 (370, 759) ^b^	1181 (865, 1505) ^c^	1215 (979, 1559) ^c^	1250–1975
EN ≥ RNI, % (n) ^2^	10.2 (68)	3.6 (6) ^a^	3.0 (5) ^a^	17.2 (29) ^b^	17.1 (28) ^b^	
Protein (g)	20 (12.8, 32.4)	16.9 (11.1, 25.7) ^a^	12.7 (8.7, 17.1) ^b^	25.3 (18.0, 37.8) ^c^	32.2 (21.9, 48.9) ^d^	
EN by Protein (%)	11 (9, 14)	12 (9, 14) ^a^	12 (11, 14) ^b^	9 (7, 12) ^c^	11 (9, 14) ^d^	10–30%
Fat (g)	19.2 (9.3, 29.1)	13.7 (7.9, 23.9) ^a^	7.2 (4.0, 13.7) ^b^	25.6 (17.4, 41.2) ^c^	24.0 (16.2, 35.1) ^c^	
EN by fat (%)	19 (13, 24)	22 (14, 29) ^a^	14 (10, 21) ^b^	20 (16, 25) ^c^	18 (13, 24) ^c^	25–35%
Carbohydrates (g)	144.1 (81.2, 218)	106.2 (68.9, 139) ^a^	66.3 (42.4, 107) ^b^	203.2 (154.8, 255) ^c^	215.2 (170.9, 270)^c^	
EN by CHO (%)	71 (64, 76)	67 (59, 76) ^a^	73.50 (66, 77) ^b^	71 (66, 75) ^c^	71 (64, 76) ^c^	45–65%
vitamin A (µg)	183 (65, 392)	253 (121, 441) ^a^	255 (158, 424) ^a^	141 (21, 456) ^b^	90 (26, 236) ^c^	450/500
RE ≥ RNI, % (n)	19.1 (127)	21.6 (36) ^a^	22.3 (37) ^a^	21.9 (37) ^a^	10.4 (17) ^b^	
Vitamin E (mg)	0.8 (0.6, 1.6)	0.9 (0.2, 2.6) ^a^	0 (0, 0.5) ^b^	0.8 (0.4, 1.3) ^c^	1.1 (0.8, 2.3) ^a^	5/7
α-TE ≥ RNI, % (n)	4.8 (32)	7.2 (12)	2.4 (4)	3 (5)	6.7 (11)	
Vitamin B1 (mg)	0.5 (0.3, 0.7)	0.5 (0.3, 0.8) ^a^	0.4 (0.3, 0.6) ^b^	0.4 (0.3, 0.6) ^b^	0.7 (0.5, 1.0) ^c^	0.6/0.9
B1 ≥ RNI, % (n)	24.9 (166)	24.0 (40) ^a^	13.9 (23) ^b^	17.2 (29) ^a,b^	45.1 (74) ^c^	
Vitamin B2 (mg)	0.5 (0.4, 0.7)	0.6 (0.4, 0.7) ^a^	0.6 (0.4, 0.8) ^a^	0.4 (0.2, 0.6) ^b^	0.5 (0.3, 0.8) ^c^	0.6/0.9
B2 ≥ RNI, % (n)	24.8 (165)	26.9 (45) ^a^	28.3 (47) ^a^	13.0 (22) ^b^	31.1 (51) ^a^	
Vitamin B6 (mg)	0.8 (0.6, 1.1)	0.7 (0.5, 0.9) ^a^	0.7 (0.5, 0.9) ^a^	0.8 (0.6, 1.0) ^b^	1.0 (0.7, 1.2) ^c^	0.6/1.0
B6 ≥ RNI, % (n)	46.2 (308)	38.9 (65) ^a^	36.7 (61) ^a^	46.7 (79) ^a^	62.8 (103) ^b^	
B12 (µg)	0 (0, 0.3)	0 (0, 0) ^a^	0 (0, 0) ^a^	0 (0, 0.9) ^b^	0.1 (0, 2.2) ^b^	1.2/1.8
B12 ≥ RNI, % (n)	14.0 (93)	6.0 (10) ^a^	1.2 (2) ^b^	21.3 (36) ^c^	27.4 (45) ^c^	
Folic acid (µg)	201 (128, 291)	206 (134, 281) ^a^	174 (116, 237) ^a^	178 (117, 285) ^a^	258 (160, 363) ^b^	200/300
FA ≥ RNI, % (n)	33.8 (225)	31.1 (52) ^a,b^	21.7 (36) ^b^	32.0 (54) ^a^	50.6 (83) ^c^	
Ascorbic acid (mg)	7.8 (1.5, 30.2)	3.3 (0.9, 9.3) ^a^	1.6 (0.7, 6.9) ^a^	15.9 (5.9, 39.1) ^b^	24.5 (9.5, 47.1) ^c^	30/35
AA ≥ RNI, % (n)	23.1 (154)	14.4 (24) ^a^	12 (20) ^a^	31.4 (53) ^b^	34.8 (57) ^b^	
Calcium (mg)	309 (133, 671)	471 (242, 801) ^a^	512 (322, 763) ^a^	155 (77, 341) ^b^	175 (86, 490) ^b^	600/700
Ca ≥ RNI, % (n)	26 (173)	32.9 (55) ^a^	35.5 (59) ^a^	13.0 (22) ^b^	22.6 (37) ^c^	
Iron (mg)	11.5 (7.6, 18.6)	15.9 (10.9, 22.7) ^a^	18.4 (13.1, 24.7) ^a^	7.2 (5.3, 9.6) ^b^	8.8 (6.2, 13) ^c^	6/9
Iron ≥ RNI, % (n)	73.7 (491)	89.2 (149) ^a^	95.8 (159) ^b^	46.7 (79) ^c^	63.4 (104) ^d^	
Zinc (mg)	4.9 (3.5, 6,5)	4.7 (3.3, 6.6) ^a^	4.4 (3.3, 5.9) ^a^	4.7 (3.5, 6.3) ^a^	5.5 (4.2, 8.1) ^b^	10.3/11.3
Zinc ≥ RNI, % (n)	4.7 (31)	4.8 (8)	4.8 (8)	1.8 (3)	7.3 (12)	
Magnesium (mg)	163 (101, 242)	186 (106, 283) ^a^	158 (96, 251) ^a,b^	141 (91, 211) ^b^	175 (121, 249) ^a^	73/100
Mg ≥ RNI, % (n)	80.3 (535)	79 (132)	78.9 (131)	76.9 (130)	86.6 (142)	

Figures are median (25th and 75th percentile) ^1^ and percentage (number) ^2^, and all such values. RE, retinol equivalent; α-TE, α-tocopherol equivalent (=vitamin E equivalent). Cut-offs for total energy requirement, adjusted for age, gender (boys and girls) and moderate levels of habitual physical activity were taken from the FAO/WHO/UNU [24]. For macronutrients, the percentage total energy intake (%EN) from proteins, total fat, and carbohydrates was evaluated using the acceptable macronutrient distribution ranges (AMDR) following the Institute of Medicine [25]. RNI/day, recommended daily nutrient intake regarding micronutrient adjusted for age (4–6, 7–9 years), and gender following the WHO recommendations [33]; for zinc, the low bioavailability was applied, and for iron, the 10% bioavailability was applied. Differences between the villages were assessed using Kruskal–Wallis and Mann–Whitney U tests for continuous variables and a Chi-square test for prevalence; all *p* values are < 0.001, except for % RE ≥ RNI = 0.013, % α-TE ≥ RNI = 0.082, % B2 ≥ RNI = 0.001, % Zinc ≥ RNI = 0.122, % Mg ≥ RNI = 0.128, and magnesium [mg] = 0.002; villages not sharing a superscript letter (^a,b,c,d^) are significantly different (*p* < 0.05) to each other.

**Table 4:** Micronutrient status and micronutrient and food intake of children compared by districts.

**Micronutrient Status**	**Chamwino, n = 333**		**Kilosa, n = 333**		***p***
Haemoglobin, g/L	123 (115, 130)		104 (111, 117)		<0.001
Anaemia, % (n)	22.2 (74)		63.7 (212)		<0.001
CRP ↑ or AGP ↑, % (n)	13.8 (46)		35.4 (118)		<0.001
Iron-ST, adj (mg/kg BW)	4.64 (3.04, 6.09)		3.67 (2.07, 5.29)		<0.001
ID adj., % (n)	19.0 (63)		39.6 (132)		<0.001
Retinol, µmol/L	0.830 (0.67, 0.97)		0.877 (0.72, 0.99)		0.028
VAD, % (n)	28.5 (95)		21.3 (71)		0.032
Zinc, mg/L	0.709 (0.64, 0.80)		0.723 (0.64, 0.80)		0.186
ZnD, % (n)	28.0 (93)		24.9 (83)		0.367
α-Carotene, µmol/L	0.038 (0.03, 0.06)		0.286 (0.18, 0.46)		<0.001
β-Carotene, µmol/L	0.526 (0.38, 0.73)		0.446 (0.31, 0.63)		<0.001
β-Cryptoxanthin, µmol/L	0.147 (0.07, 0.36)		0.173 (0.07, 0.32)		0.841
Lutein-zeaxanthin, µmol/L	1.176 (0.94, 1.51)		0.669 (0.50, 0.89)		<0.001
Lycopene, µmol/L	0.227 (0.15, 0.34)		0.531 (0.33, 0.75)		<0.001
γ-Tocopherol, µmol/L	1.145 (0.82, 1.66)		0.444 (0.32, 0.64)		<0.001
α-Tocopherol, µmol/L	14.15 (12.3, 16.1)		16.86 (14.7, 19.6)		<0.001
**Micronutrient intake**					
Iron intake, mg	17.2 (12.2, 24.3)		8.0 (5.8, 11.0)		<0.001
Iron suff. % (n)	92.2 (317)		55.0 (183)		<0.001
RE intake, µg	255 (151, 438)		104 (23, 308)		<0.001
RE suff. % (n)	21.9 (73)		16.2 (54)		0.061
Zinc intake, mg	4.49 (3.32, 6.19)		5.19 (3.76, 7.1)		0.006
Zinc suff. % (n)	4.8 (16)		4.5 (15)		0.854
α-TE intake, mg	0.32 (0.02, 1.60)		0.77 (0.45, 1.51)		<0.001
α-TE suff., % (n)	4.8 (16)		4.8 (16)		1.000
**Food Intake**	**% (N)**	**Grams**	**% (N)**	**Grams**	***p***
Millet ^1^	92 (307)	375 (250, 500) *	4 (14)	250 (181, 375)	<0.001
Maize ^2^	11 (36)	250 (190, 500)	86 (288)	250 (131, 400)	<0.001
Rice (with oil) ^3^	3 (11)	205 (125, 300)	61 (203)	250 (250, 300)	<0.001
DGLV ^4^	92 (305)	126 (90, 219)	31 (104)	100 (63, 150)	<0.001
Vegetables ^5^	8 (26)	95 (49, 131)	27 (90)	100 (50, 125)	<0.001
Legumes ^6^	53 (177)	20 (10, 93)	77 (258)	125 (100, 211) **	<0.001
Roots ^7^	1 (5)	83 (32, 106)	26 (88)	168 (100, 200) *	<0.001
Fruits ^8^	10 (35)	153 (115, 200)	18 (59)	182 (100, 300)	0.008
Meat ^9^	4 (14)	75 (49, 115)	14 (47)	75 (50, 100)	<0.001
Fish ^10^	2 (8)	95 (49, 107)	25 (85)	125 (72, 150)	<0.001

Data are median (25th and 75th percentile) or percentage (number); *p*-value: Mann–Whitney U test for continuous variables, Chi-square test for prevalence (e.g., % MN deficiency); * *p* < 0.05; ** *p* < 0.001 for food intake data in grams (Mann–Whitney U test); N = 332/333 serum samples in Chamwino. CRP ↑ or AGP ↑, CRP > 5 µg/L or AGP > 1 g/L. Iron-ST adj., total body iron stores adjusted (for ferritin); ID adj., iron deficiency if serum ferritin (adjusted) < 15 µg/L or sTfR > 8.5 mg/L. ^1^ Millet includes pearl and finger millet dishes; ^2^ maize includes stiff porridge ‘Ugali’ and soft porridge dishes; ^3^ rice cooked with coconut or oil and onions; ^4^ dark green leafy vegetables (DGLV) include ‘Mlenda’ or ‘Ilende’, amaranth, cow pea, sweet potato, and pumpkin leaves or spinach; ^5^ vegetables: okra, pumpkin, tomato, African eggplant, Chinese, or white cabbage; ^6^ legumes include beans, peas, bambara nut, and ground nut; ^7^ roots include cassava, potatoes, and yams; ^8^ fruits include banana, baobab, guava, mango, papaya, and water melon; ^9^ meat includes beef, goat, chicken, and pork; ^10^ fish includes fish relish and dried sardines.

**Table 5:** Determinants of stunting, underweight, and overweight in Chamwino and Kilosa district.

		Chamwino			Kilosa	
	HAZ < −2SD (25.5%) N = 85	WAZ < −2SD (15.3) N = 51	BMI > 1SD (2.7%) N = 9	HAZ < −2SD (30.6%) N = 102	WAZ < −2SD (13.5%) N = 45	BMI > 1SD (7.5%) N = 25
Age, years	0.71 (0.54, 0.94)	2.94 (1.77, 4.88) *	0.36 (0.14, 0.97)		1.93 (1.26, 2.98)	0.32 (0.17, 0.60) *
WAZ < −2SD	23.8 (10.7, 53.0) *			29.2 (11.0, 77.0) *		
HAZ < −2SD		30.5 (10.7, 86.5) *			20.4 (6.97, 58.9) *	
MUAC, cm		0.11 (0.05, 0.23) *	7.87 (3.10, 20.0) *		0.18 (0.10, 0.33) *	7.20 (3.74, 13.8) *
ID adj., =1 (yes)						4.53 (1.37, 15.0)
VAD, =1 (yes)	2.09 (1.12, 3.90)					
ZnD, =1 (yes)			6.46 (0.99, 42.18)			
β-Cryptoxanthin, µmol/L		0.10 (0.01, 0.75)				
Lutein-zeaxanth., µmol/L						5.91 (1.32, 26.5)
B6 intake ≥ RNI, =1						12.3 (2.24, 68.0)
R^2^ (Nagelkerke)	0.369	0.640	0.546	0.298	0.613	0.576

Multiple logistic regression models with a forward approach; values are Exp (B) = odds ratio, and 95% CI; all factors at *p* < 0.05, except * *p* < 0.001. ID adj. = 1: iron deficiency (ferritin adjusted), yes vs. no = 0; VAD = 1: vitamin A deficiency, yes vs. no = 0; ZnD = 1: zinc deficiency, vs. no = 0. Variables in the initial models included age, sex, reported malaria (in the last 3 months) or diarrhoea (in the last 4 weeks), family size (number of persons in the HH), mother’s literature status (can read and write = 1), HAZ < −2SD, WAZ < −2SD, anaemia (=1), MN deficiencies: ID, VAD, ZnD, serum micronutrients (e.g., β-carotene), and sufficient dietary intakes of assessed MNs (e.g., iron or vitamins ≥ RNI). HAZ < −2SD, stunting; WAZ < −2SD, underweight; BMI > 1SD, overweight; VAD = 1, vitamin A deficiency, ID = 1, iron deficiency, ZnD = 1, zinc deficiency.

**Table 6:** Determinants of anaemia, iron deficiency (ID), and vitamin A deficiency (VAD) in Chamwino and Kilosa districts.

		Chamwino			Kilosa	
	Anaemia (22.3%) N = 74	ID (19.0%) N = 63	VAD (28.5%) N = 95	Anaemia (63.7%) N = 212	ID (39.6%) N = 132	VAD (21.3%) N = 71
Malaria, =1		2.43 (1.28, 4.62)				
High CRP or AGP, =1			4.02 (1.95, 8.27) *	2.02 (1.22, 3.34)	1.84 (1.17, 2.91)	2.54 (1.43, 4.52)
ID, =1 (yes)	1.90 (1.00, 3.64)		2.39 (1.29, 4.44)			
VAD, =1 (yes)	2.05 (1.16, 3.62)	2.79 (1.57, 4.97) *				
ZnD, =1 (yes)	2.19 (1.26, 3.80)					2.71 (1.53, 4.81)
β-Carotene, µmol/L			0.29 (0.10, 0.88)			
β-Cryptoxanthin, µmol/L			0.26 (0.07, 0.93)			
α-Tocopherol µmol/L			0.88 (0.80, 0.96)			0.88 (0.80, 0.96)
Lycopene, µmol/l				0.35 (0.17, 0.71)		
Lutein-zeaxanth., µmol/L						0.30 (0.10, 0.86)
B6 intake ≥RNI, =1	2.05 (1.19, 3.55)					
R^2^ (Nagelkerke)	0.126	0.097	0.204	0.073	0.028	0.195

Multiple logistic regression models with a forward approach; values are Exp (B) = odds ratio (95% CI; all factors at *p* < 0.05, except * *p* < 0.001. Variables in the initial models included age, sex, reported malaria (in the last 3 months) or diarrhoea (in the last 4 weeks), family size (number of persons in the HH), mother’s literature status (can read and write = 1), MN deficiencies (ID = 1, iron deficiency (vs. no = 0); VAD = 1, vitamin A deficiency (vs. no = 0); ZnD = 1, zinc deficiency (vs. no = 0)), serum micronutrients (e.g., β-carotene), and sufficient dietary intakes of assessed MNs (e.g., iron or vitamins ≥ RNI).
